# Effect of Storage Time and Temperature on the Bioactivity of a Chitosan-Derived Epigenetic Modulation Scaffold

**DOI:** 10.3390/md21030175

**Published:** 2023-03-12

**Authors:** Teerawat Sukpaita, Suwabun Chirachanchai, Atiphan Pimkhaokham, Ruchanee Salingcarnboriboon Ampornaramveth

**Affiliations:** 1 Center of Excellence on Oral Microbiology and Immunology, Faculty of Dentistry, Chulalongkorn University, Bangkok 10330, Thailand; 2 Department of Oral Surgery, Faculty of Dentistry, Naresuan University, Phitsanulok 65000, Thailand; 3 Bioresources Advanced Materials (B2A), The Petroleum and Petrochemical College, Chulalongkorn University, Bangkok 10330, Thailand; 4 Center of Excellence on Petrochemical and Materials Technology, Chulalongkorn University, Bangkok 10330, Thailand; 5 Department of Oral and Maxillofacial Surgery, Faculty of Dentistry, Chulalongkorn University, Bangkok 10330, Thailand; 6 Department of Microbiology, Faculty of Dentistry, Chulalongkorn University, Bangkok 10330, Thailand

**Keywords:** storage, temperature, epigenetic, trichostatin A, chitosan, bone scaffold

## Abstract

The appropriate storage protocol is one of the main limitations of translating tissue engineering technology to commercialized clinical applications. Recently, the development of a chitosan-derived composite scaffold incorporated with bioactive molecules has been reported as an excellent material to repair a critical size bony defect in mice calvaria. This study aims to determine the storage time and appropriate storage temperature of Chitosan/Biphasic Calcium Phosphate/Trichostatin A composite scaffold (CS/BCP/TSA scaffold) in vitro. The mechanical properties and in vitro bioactivity of trichostatin A (TSA) released from CS/BCP/TSA scaffolds in different storage times and temperatures were evaluated. Different storage times (0, 14, and 28 days) and temperatures (−18, 4, and 25 °C) did not affect the porosity, compressive strength, shape memory, and amount of TSA released. However, scaffolds stored at 25 °C and 4 °C were found to lose their bioactivity after 3- and 7-day storage periods, respectively. Thus, the CS/BCP/TSA scaffold should be stored in freezing conditions to preserve the long-term stability of TSA.

## 1. Introduction

The human bone is a complex hierarchical tissue that regenerates itself after injury. However, the spontaneous skeletal defect repair by the physiological bone-remodeling process is not always ideal [1]. Bone grafts are substituted materials to fill or repair bone defects [2]. Besides mechanical support, grafting material promotes bone healing due to its osteoconductive and osteoinductive properties [3]. Generally, bone grafts and substitutes are attempted to facilitate and enhance healing [4]. Several types of bone grafts have been introduced. Nevertheless, the ideal material is yet to be determined [5].

The most compatible bone substitute is an autogenous bone graft, a gold standard for bony defect repair [6]. However, this grafting technique has several limitations, especially its availability and the requirement to perform a second surgery site. As an alternative, other bone grafts, such as allografts, xenografts, and alloplastic materials, have been developed to replace autografts [7]. However, compared to autologous bone, the disadvantages are limited regenerative properties, tissue compatibility, and risk of infectious disease transmission [8]. To overcome these limitations, scaffold-based biomaterials for tissue-engineered could serve as an alternative treatment for maxillofacial bone tissue regeneration [9].

Bone tissue engineering (BTE) aims to develop bone scaffolds with applicable mechanical properties and suitable porosity to support osteoblast proliferation and differentiation [10]. Chitosan-based biomaterials have been introduced as bone scaffolds due to their biocompatible, biodegradable, and osteoconductive properties [11,12,13,14]. TSA is a potent and reversible histone deacetylases inhibitor, which induces hyper-acetylation of histone proteins in mammalian cells [15]. Additionally, TSA has been reported to promote mesenchymal stem cells (MSCs) to differentiate into osteoblasts [16] and increase bone regenerative capacity [17]. All these findings indicate that TSA is a promising candidate as an osteogenic-inducing molecule for BTE.

Our recent research developed a chitosan-based scaffold loaded with TSA, which promoted osteogenesis and exhibited a sustained release of active TSA. The scaffold with 800 nM of TSA could increase RUNX2 activity in the primary human periodontal ligament cells (hPDLCs). This composite scaffold demonstrated remarkable biocompatibility and induced robust bone formation in the critical mouse calvarial defect [18]. Besides excellent physiological and biological properties, the stability of the biomaterial over time is an essential factor for its clinical application. It was hypothesized that changing the storage condition could affect the long-term mechanical properties and biological functions of the CS/BCP/TSA scaffold. In this study, we plan to evaluate the shelf-life and appropriate storage temperature of the fabricated CS/BCP/TSA composite scaffold.

## 2. Results

### 2.1. Microstructure and Porosity of the Stored Scaffold

**Figure 1:**
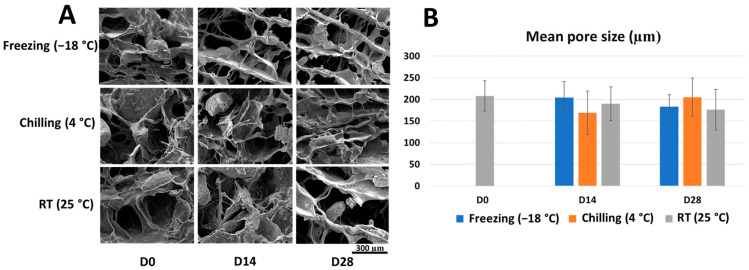
Microstructure and porosity of stored scaffold. (**A**) SEM microphotographs of CS/BCP/TSA scaffolds in various storage conditions. (**B**) Mean pore size of CS/BCP/TSA scaffolds in various storage conditions. The results are presented as the mean ± standard deviation.

The SEM micrographs of the CS/BCP/TSA scaffolds in each storage condition are shown in Figure 1A. The scaffolds show very similar interconnected porous structures with the rough surface of BCP particles in all storage conditions at every time point. The representative pore size distribution of all storage conditions was in the range of 100–250 μm. At day 14, the average pore size was determined as 204.08 ± 37.02 μm, 169.12 ± 50.71 μm, and 190.32 ± 39.23 μm in freezing, chilling, and room temperature (RT) conditions, respectively. At day 28, the samples showed a similar pattern, with the average pore size being 183.21 ± 28.042 μm, 205.89 ± 44.03 μm, and 176.62 ± 47.31 μm in freezing, chilling, and RT conditions, respectively. The Student’s *t*-test indicated no significant difference in the mean pore size of all storage conditions at 14 and 28 days compared to the freshly fabricated scaffold (Figure 1B).

### 2.2. Mechanical Strength and Shape Memory Ability of the Stored Scaffold

**Figure 2:**
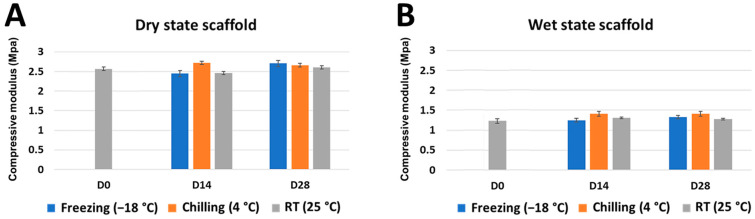
Compression testing graph of the CS/BCP/TSA scaffolds in various storage conditions. (**A**) Dry state scaffold and (**B**) wet state scaffold; the results are presented as the mean ± standard deviation.

The summary of the mechanical strength results is shown in Figure 2. Scaffold in a dry state at day 14 has a compressive modulus of 2.443 ± 0.03 megapascal (MPa), 2.717 ± 0.02 MPa, and 2.456 ± 0.02 MPa in freezing, chilling, and RT conditions, respectively. The compressive modulus of the scaffolds in the dry stage at day 28 was 2.701 ± 0.02 MPa, 2.658 ± 0.02 MPa, and 2.60 ± 0.02 MPa in freezing, chilling, and RT conditions, respectively. There was no significant change in the compressive modulus of the scaffolds regardless of the storage time (days 0, 14, and 28).

The strength of the scaffold in the wet stages was decreased when compared to the dry state. The compressive modulus was in the range of 1–1.5 MPa. However, the analysis showed no statistical difference in the scaffolds’ compressive strength with varying storage temperatures compared to the freshly fabricated scaffold (Student’s *t*-test).

**Figure 3:**
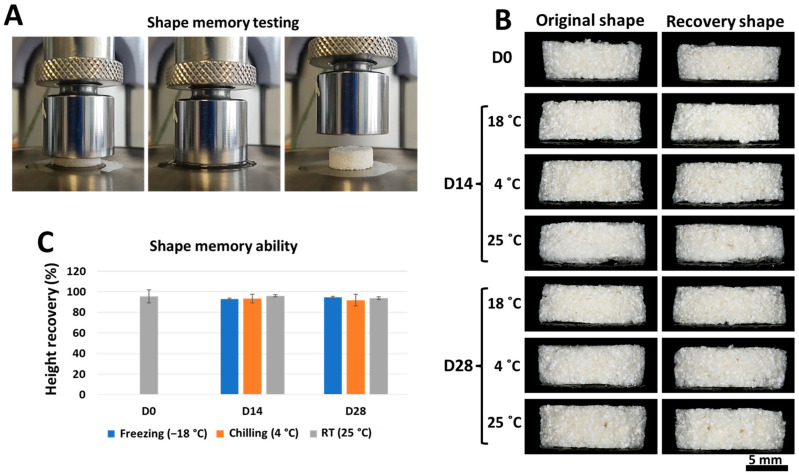
Shape memory ability of the CS/BCP/TSA scaffolds in various storage conditions. (**A**) Testing steps: wet state scaffold was compressed using UTM, the compression was held for 300 s, and the scaffolds were left to recover for 24 h. (**B**) Gross structure images of the CS/BCP/TSA scaffolds before and after compression. (**C**) Percentage of height recovery of the CS/BCP/TSA scaffolds in various storage conditions.

Similar to other mechanical properties, the effect of storage conditions on the shape memory of the scaffolds showed no significant difference in any of the samples on both days 14 and 28. All samples were above 90% height recovery between 91.87 and 96.1% (Figure 3B,C). The shape recovery of the scaffolds was also demonstrated in wet conditions, as shown in Figure 3A.

### 2.3. Release and Bioactivity of TSA from the Stored Scaffold

**Figure 4:**
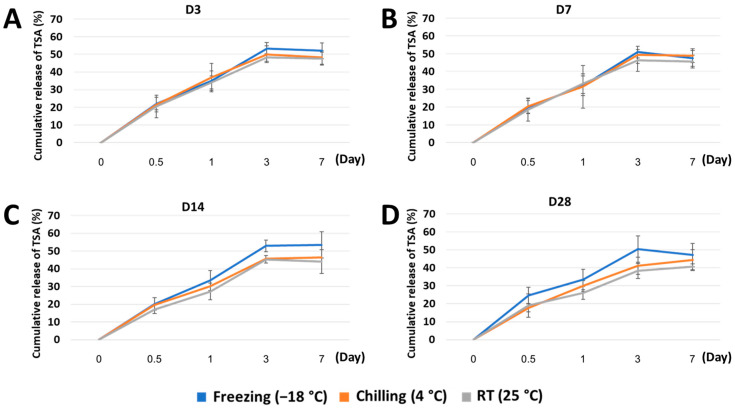
Cumulative release curve of TSA from the CS/BCP/TSA scaffolds in various storage conditions: (**A**) 3 days, (**B**) 7 days, (**C**) 14 days, and (**D**) 28 days.

The effect of storage conditions on in vitro release of TSA from the stored scaffold was measured using an ultraviolet–visible spectrophotometer. Figure 4 shows a linear increase of the TSA cumulative release of approximately 50% of initial loading within 3 days. This phenomenon was seen in all storage conditions at all predetermined time points. The data did not indicate significant differences in TSA cumulative release among each storage condition.

**Figure 5:**
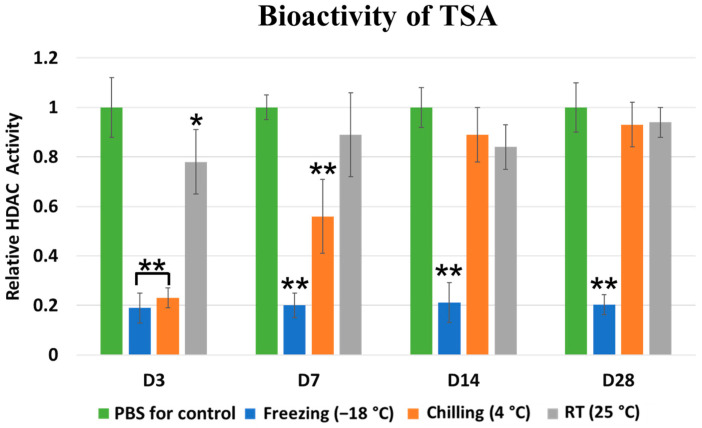
Bioactivity of TSA released from the CS/BCP/TSA scaffolds in various storage conditions. *, ** indicates statistically significant difference at *p* < 0.05, *p* < 0.01, respectively, when compare with control.

The results of TSA bioactivity were demonstrated as a reduction in the relative HDAC activity, as shown in Figure 5. In contrast to the releasing test, storage time and temperature significantly affect the TSA bioactivity. In the case of the 3-day storage period, the freezing and chilling storage conditions showed very strong TSA bioactivity when compared to the negative control. In contrast, the RT condition showed a partially decreasing TSA bioactivity. On the contrary, the TSA bioactivity under chilling storage conditions was slightly reduced at a 7-day storage period. Moreover, the bioactivity was completely lost in the RT storage on days 14 and 28. TSA bioactivity was observed in freezing storage conditions at all time points. This result showed that freezing storage conditions could preserve TSA activity for up to 28 days.

## 3. Discussion

### 3.1. An Appropriate Storage Protocol Is an Important Key for Commercialized Clinical Applications of the CS/BCP/TSA Scaffold

Prior work has demonstrated the CS/BCP/TSA scaffold is an up-and-coming candidate for bone substitution material owing to its osteoinductive ability. This epigenetic modulation scaffold has advantageous characteristics, including (a) excellent biocompatibility with human cells, (b) induced robust osteoblast-related gene expression, and (c) excellent bone regeneration ability in a mouse calvarial defect model [18]. The details of chitosan-derived epigenetic modulation scaffold in comparison to other commercially available growth factor-enhanced bone grafts are shown in Table 1, adapted with permission from [19], 2021, Marco Govoni.

Although a significant amount of research has recently been conducted on scaffold development, one of the main limitations of commercializing tissue-engineered products is the lack of a standard storage protocol to preserve long-term mechanical properties and biological functions [20]. Moreover, optimal storage time and temperature guarantee continuous supply for clinical applications [21]. Various storage conditions have been used to preserve tissue-engineered products [22]. The European Pharmacopoeia (Pharm. Eur.) gives the most used medical device preservation approaches, including (a) freezing, which uses temperatures well below the freezing point, conventionally below minus 15 °C (5 °F); (b) chilling, the application of temperatures in the range of 2–8 °C (36–46 °F); (c) RT, the application of temperatures in the range of 15–25°C (59–77 °F) [23,24].

This work focused on the evaluation of in vitro mechanical property maintenance, including surface morphology, compressive modulus and shape memory ability of the CS/BCP/TSA scaffolds, and bioactivity of TSA that released from the scaffolds in different temperature storage including freezing conditions (−18 °C), chilling conditions (4 °C), and RT conditions (25 °C) for up to 28 days.

### 3.2. Different Storage Times and Temperatures Did Not Affect the Scaffolds’ Mechanical Properties

BTE requires a suitable surface morphology for the porous scaffold [25]. The average pore size of tissue-engineered scaffold considerably affects the in-growth of tissue and vasculature as well as the proliferation and differentiation of stem cells [26]. The interconnected pores diameter over 100 μm with a high degree of distribution are generally preferred for osteogenic stem cell migration and bone tissue ingrowth [27]. The SEM results proved that the surface morphology of the CS/BCP/TSA scaffolds matched the ideal scaffold requirement. Moreover, the scaffolds’ porosity evaluation showed no significant difference in all storage conditions. This result demonstrated that the different storage conditions did not affect the architecture of the scaffolds.

In addition to the geometry, the optimal compressive modulus of the BTE scaffold is achieved when given in the 1–12 MPa range, matching the bearable range of trabecular bone [28]. In this study, the compressive strength range for all the wet-state scaffolds was 1–1.5 MPa, which showed matchable mechanical properties with the scaffold requirement. All stored samples exhibit similar mechanical strength with no statistical difference related to their porous microstructures. This result demonstrated that the different storage times and temperatures did not affect the scaffolds’ mechanical strength.

Another critical ability of tissue-engineered scaffolds is the shape memory effect. This property allows the fabricated scaffold to be press-fitted into irregularly shaped bone defects [29]. The height recovery measurement data showed that CS/BCP/TSA scaffolds possess excellent shape memory effects with >90% of the original height. This result proved that the three-dimensional CS/BCP/TSA scaffolds were a smart memory material, and the different storage conditions did not affect their ability. The shape memory results corresponded with the previous studies that chitosan-based scaffolds possess excellent shape recovery effects [30,31]. This phenomenon might be because the chitosan molecules work as strong crosslinking nodes, which can pull back the polymer structure to its original shape [32].

These observations align with the mechanical properties of the CS/BCP/TSA scaffold, as described by our previous studies [18,33]. Interestingly, all storage conditions, including freezing at −18 °C, chilling at 4 °C, and room temperature at 25 °C, did not affect the scaffolds’ mechanical properties, even if stored for up to 28 days. These experimental data corresponded with a previous report; chitosan can maintain its hardness and mechanical strength at a temperature below 40 °C [34]. This evidence demonstrated that chitosan-based biomaterials could be stored long-term at room temperature.

### 3.3. Released TSA Lost Its Activity When Scaffolds Were Stored at RT or Chilling Temperature

It is also known that TSA, a histone deacetylase (HDAC) inhibitor, can promote osteoblast differentiation through the modulation of the ERK 1/2 signaling pathways [35]. Huynh et al. reported that TSA induces hyper-acetylation of Histone H3, a key molecule involved in epigenetic mechanisms. In addition, TSA can also enhance osteogenesis by increasing RUNX2 expression in osteoblasts [16,17].

Our recent study developed a chitosan-based scaffold incorporated with TSA, and this epigenetic modulation scaffold exhibited favorable TSA released for up to 3 days [18]. Despite its excellent in vitro and in vivo bone regeneration ability, using TSA as an osteogenic molecule has significant challenges in terms of drug stability. The manufacturer recommended keeping TSA at freezing temperature (−18 °C) for activity preservation. Interestingly, the UV–Vis spectrophotometer detected the accumulation of TSA molecules in the conditioned media of freeze and chilled scaffolds, similar to the newly constructed scaffold. However, the HDAC activity analysis showed a significant decrease in TSA activity of the scaffold stored at RT and chilling temperature at 3 and 7 days, respectively. This result demonstrated that the released TSA might lose its inhibitory property in long-term storage at RT and chilling temperature. A possible explanation might be that the increasing temperature affect the TSA’s zinc-binding tail but not the TSA’s aromatic ring structure, which a spectrophotometer can detect.

Overall, the results of this study demonstrated that freezing temperature is better than chilling temperature and RT for the long-term storage of the CS/BCP/TSA scaffold due to the preservation of TSA bioactivity. The freezing method has been recommended for storing some biomaterials, but it is not widely used for bone substitute material storage due to transportation difficulty. It has been evidenced that the microsphere release system can improve the efficacy and stability of drugs [36,37,38,39]. Further studies should be conducted to encapsulate TSA molecules into micro- or nano-particles before loading the scaffold, resulting in the long-term stability of TSA in high-temperature storage.

## 4. Materials and Methods

### 4.1. Fabrication of CS/BCP/TSA Scaffold

Based on our previous reports, porous CS/BCP/TSA scaffolds were prepared via a freeze-drying technique [18,33,40]. Chitosan powder with ≥90% deacetylation, Mw: 250 kDa, Particle size: 0.5 mm, was obtained from Marine BioResources, Thailand. Then, 4% *w*/*v* of CS solution was prepared by dissolving in succinic acid solution on a stirrer with a hot mantle (60 °C) for 12 h. Subsequently, 20% *w*/*w* of BCP (MTEC, Pathumthani, Thailand, and TSA (Sigma-Aldrich, Oakville, Canada) 800 nM were added to the flask, and stirring was maintained at 100 rpm. The 1-(3 dimethylamino-propyl)-3 ethyl carbodiimide hydrochloride (EDC) and N-Hydroxysuccinimide (NHS) solution were used as the crosslinking agents. The hydrogel-based scaffolds were filled into the cylindrical polypropylene mold with a 10 mm diameter. Finally, the CS/BCP/TSA hydrogels were freeze-dried at −50°C for 48 h to obtain the porous CS/BCP/TSA scaffold.

### 4.2. Storage Protocol

**Figure 6:**
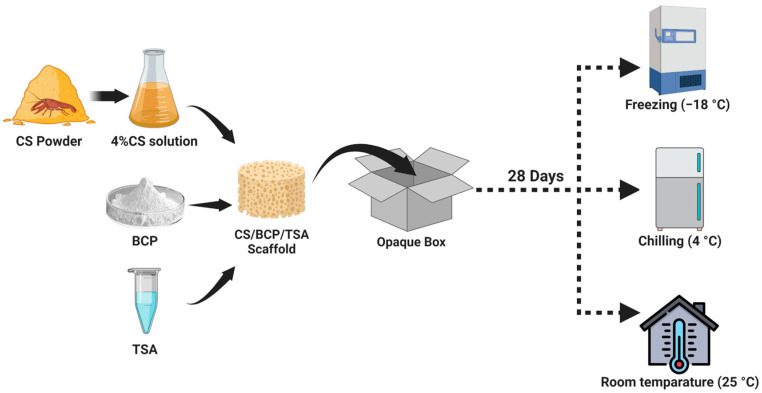
Fabrication and storage protocol of the CS/BCP/TSA scaffold.

A cylindrical CS/BCP/TSA scaffold, 10 mm in diameter and 5 mm in height, was used in further testing. The fabricated scaffolds were immediately stored in the sealed opaque high-density polyethylene (HDPE) box and randomly divided into three groups of different storage temperatures: (1) freezing conditions at −18 °C, (2) chilling conditions at 4 °C, (3) at room temperature (RT) at 25 °C. The stored scaffolds were collected for further evaluation for 0, 14, and up to 28 days. The schematic illustration of the fabrication and storage protocol is shown in Figure 6.

### 4.3. Surface Morphologies

The porous microstructures of scaffolds were observed using scanning electron microscopy (SEM). Scaffolds were mounted on aluminum plates, then the cross-section surface of the scaffolds was coated with gold, and the surface morphologies of each storage condition of CS/BCP/TSA scaffolds were evaluated by image visualization software (Image J 1.53, NIH, Bethesda, MA, USA). The pore size of the fabricated scaffolds was measured on a random 1 cm^2^ area of the SEM images based on 100 pores, and reported as mean pore size.

### 4.4. Compressive Mechanical Properties

Compression tests of the CS/BCP/TSA scaffolds in dry and wet states were performed using an Instron Model 55R4502 universal testing machine (UTM) (Instron, Kawasaki, Japan) with 100 N load cells. For the wet state, scaffolds were immersed in PBS solution at 37 °C for 1 h before measurement. The 1 mm/min loading rate was applied until the scaffold was compressed to approximately 80% of its original. The average compressive modulus was calculated from the slope of the initial linear area of the stress–strain curve. All conditions were measured for 3 samples (*n* = 3).

### 4.5. Shape Memory Testing

To demonstrate the shape recovery ability of the CS/BCP/TSA scaffolds, the fabricated cylindrical scaffolds (10 × 5 mm^3^) were imaged horizontally, and the height was measured using Image J software (NIH, version 1.53, Bethesda, MD, USA) at the left, middle, and right edge of the scaffolds. To mimic the clinical situation, the scaffolds were humidified in PBS at 37 °C for 1 h. The UTM (Instron, Kawasaki, Japan) with 100 N load cells was used for the scaffold compressing (80% of their original high) for 300 s. Then, the compressed scaffolds were stored in a sealed box at 25 °C for 24 h, and the percentage of height recovery by the time-lapsed picture was used for shape memory analysis.

### 4.6. Culture of Primary Human Periodontal Ligament Cells

With informed consent, the human cell culture experiment was performed in accordance with the Declaration of Helsinki. The primary human periodontal ligament cells (hPDLCs) were extracted from the molars of healthy individuals. Approval for this current study was obtained from the Ethical Committee of the Faculty of Dentistry, Chulalongkorn University, Thailand (Approval Number: HREC-DCU 2020-106). Following extraction, the periodontal ligament tissue was cut from the middle one-third of the root surface using aseptic techniques. Explants were cultured using culture medium (10% FBS, 1% L-Glutamine, 1% antibiotics in DMEM, Gibco) and incubated at 37 °C until outgrowing cells reached confluence.

### 4.7. In Vitro Release and Bioactivity of TSA

In vitro release of TSA was studied by incubating CS/BCP/TSA scaffolds in 25 mL of PBS inside a 37 °C incubator for 1 h. Subsequently, as indicated, 1 mL of incubated solution at different time intervals was analyzed using a UV–Vis spectrophotometer at 280 nm. A standard curve of TSA concentration up to 800 nM was created. The cumulative release of TSA in the respective samples was calculated and expressed as a percentage of the initial loading.

The bioactivity of the released TSA was evaluated using a fluorometric HDAC Activity Assay kit (Abcam, Cambridge, UK). The extracted hPDLCs were incubated with 1 mL of the collected solution of the scaffold at 37 °C for 24 h. Subsequently, the cell lysates were incubated with a buffer containing a substrate peptide for 30 min at 37 °C. Then, developing reagent was added for a further 20 min. The fluorescence intensity at Ex/Em = 350/460 nm was evaluated. The bioactivity of TSA in the respective samples was calculated and expressed as a relative HDAC activity of hPDLCs.

### 4.8. Statistical Test Methods

Each experiment was conducted in triplicate to confirm its reproducibility. Statistical evaluation was performed with SPSS 23 Statistics (IBM, Armonk, NY, USA). The experimental results were described as the mean and SD. One-way ANOVA and the Student’s *t*-test were used to determine statistical significance.

## 5. Conclusions

Storage time and temperature did not affect the porosity, compressive strength, shape memory ability, and TSA release of the CS/BCP/TSA scaffold. However, scaffolds stored at a chilled temperature of 4 °C and at 25 °C room temperature were found to lose their TSA activity after 7 days. Thus, the CS/BCP/TSA scaffold should be stored in freezing conditions to preserve the long-term stability of TSA.

## Figures and Tables

**Table 1:** The components and applications of chitosan-derived epigenetic modulation scaffold in comparison to other commercially available growth factor-enhanced bone grafts.

Material or Commercial Name	Osteoinductive Molecule	Scaffold Carrier	Performance and Applications
CS/BCP/TSA scaffold	TSA	Chitosan/Biphasic Calcium Phosphate	Maxillo-facial bone augmentations Alveolar ridge preservation
INFUSE^®^	Recombinant human bone morphogenetic protein-2 (rhBMP-2)	Collagen sponge	Maxillo-facial bone augmentations Spinal fusion procedures
OsteoAMP^®^	Allogeneic morphogenetic protein (AMP)	Allograft	Spinal fusion procedures
Augment^®^	Recombinant human platelet-derived growth factor-BB (rhPDGF-BB)	β-Tricalcium phosphate	Foot or ankle fusion procedures

## Data Availability

Not applicable.
